# Supplementary material for: Variability of cost trajectories over the last year of life in patients with advanced breast cancer in the Netherlands
Source: PLoS One. 2020 Apr 9;15(4):e0230909. doi: 10.1371/journal.pone.0230909 (PMC7145011; doi:10.1371/journal.pone.0230909)
Supplement: S1 Table — (DOCX) [file pone.0230909.s001.docx]

**Appendix**

**Supporting information for: “Variability of cost trajectories over the last year of life in patients with advanced breast cancer in the Netherlands”**

| **S1 Table: Unit costs (in €2017)** | | | | | | | | | | | | |  |
| --- | --- | --- | --- | --- | --- | --- | --- | --- | --- | --- | --- | --- | --- |
| **Resource** | | **Unit cost** | | **Ref** | | **Resource** | | **Unit cost** | | **Ref** | | |  |
| 5-fluorouracil | | 0.0067 | | 3 | | Docetaxel | | 5.04 | | 3 | | |  |
| Abdominal surgery | | Confidential | | 2 | | Doxorubicin | | 22.02 | | 3 | | |  |
| Ablatio with reconstruction | | Confidential | | 2 | | Drainage of ureter | | Confidential | | 2 | | |  |
| Alendronic acid | | 0.01 | | 3 | | Drug administration | | 130.00 | | 4 | | |  |
| Anastrozol | | 0.03 | | 3 | | Echocardiography | | 78.78 | | 1 | | |  |
| Ascites drainage | | 143.24 | | 1 | | Emergency Dep. visit | | 263.40 | | 1 | | |  |
| Axillary node dissection | | Confidential | | 2 | | Epirubicine | | 6.15 | | 3 | | |  |
| Bevacizumab | | 3.37 | | 3 | | Eribulin | | 480.19 | | 3 | | |  |
| Biopsy | | Confidential | | 2 | | Erythrocytes transfusion | | 219.67 | | 1 | | |  |
| Bone scan | | Confidential | | 2 | | ER-test | | 47.40 | | 5 | | |  |
| Brain/neurosurgery | | Confidential | | 2 | | Etoposide | | 0.17 | | 3 | | |  |
| Brivanib | | 1.00 | | 3 | | Everolimus | | 14.58 | | 3 | | |  |
| CA 15.3 | | Confidential | | 2 | | Exemestane | | 0.0092 | | 3 | | |  |
| Capecitabin | | 0.0027 | | 3 | | Fulvestrant | | 1.30 | | 3 | | |  |
| Carboplatin | | 0.31 | | 3 | | Gemcitabin | | 0.09 | | 3 | | |  |
| Central venous catheter | | Confidential | | 2 | | Gosereline | | 340.58 | | 3 | | |  |
| Clodronic acid | | 0.0041 | | 3 | | HER2 FISH test | | 148.13 | | 5 | | |  |
| CT abdomen | | 192.35 | | 1 | | HER2 IHC | | 47.40 | | 5 | | |  |
| CT other | | 131.98 | | 1 | | Hickmann | | Confidential | | 2 | | |  |
| CT thorax | | 185.19 | | 1 | | Ibandronic acid | | 0.11 | | 3 | | |  |
| CT thorax abdomen | | 377.54 | | 1 | | ICU in-patient day | | 1,206.16 | | 1 | | |  |
| Cyclophosphamide | | 0.03 | | 3 | | In-patient day | | 646.81 | | 1 | | |  |
| Cytarabine | | 1.33 | | 3 | | Lapatinib | | 0.07 | | 3 | | |  |
| Denosumab | | 3.58 | | 3 | | Letrozole | | 0.02 | | 3 | | |  |
| DEXA Bone Densitometry | | 78.78 | | 1 | | Liver surgery | | Confidential | | 2 | | |  |
| **Table S1 (continued): Unit costs (in 2017 €)** | | | | | | | | | | | | | |
| **Resource** | | | **Unit cost** | | **Ref** | | **Resource** | | **Unit cost** | | **Ref** | | |
| Lumpectomy | | | Confidential | | 2 | | Port a cath | | Confidential | | 2 | | |
| Mamma sono | | | 89.01 | | 1 | | PR-test | | 47.40 | | 5 | | |
| Mastectomy | | | Confidential | | 2 | | Pulmonary surgery | | Confidential | | 2 | | |
| Medroxyprogesteron | | | 0.0051 | | 3 | | Radiotherapy | | 252.81 | | 6 | | |
| Megestrol | | | 0.02 | | 3 | | Resection metastasis (other) | | Confidential | | 2 | | |
| Methotrexate | | | 0.23 | | 3 | | Resection of skin metastasis | | Confidential | | 2 | | |
| Mitomycin | | | 3.86 | | 3 | | Risedronic acid | | 0.02 | | 3 | | |
| MRI | | | 257.83 | | 1 | | Secondary reconstruction | | Confidential | | 2 | | |
| MUGA scan | | | 78.78 | | 1 | | Sentinel node procedure | | Confidential | | 2 | | |
| Olaparib | | | 0.24 | | 3 | | Stent bile duct | | Confidential | | 2 | | |
| Orthopedic surgery: extremities | | | Confidential | | 2 | | Stent gastro-intestinal tract | | Confidential | | 2 | | |
| Orthopedic surgery: spine | | | Confidential | | 2 | | Surgery contralateral axilla | | Confidential | | 2 | | |
| Paclitaxel | | | 2.49 | | 3 | | Surgery of nodes (outside) | | Confidential | | 2 | | |
| Palbociclib | | | 1.64 | | 3 | | T-DM1 | | 18.66 | | 3 | | |
| Pamidronic acid | | | 2.02 | | 3 | | Tamoxifen | | 0.01 | | 3 | | |
| Pertuzumab | | | 0.17 | | 3 | | Telephone consultation | | 17.29 | | 1 | | |
| PET and MRI Scan | | | Confidential | | 2 | | Thrombocytes transfusion | | 530.87 | | 1 | | |
| PET low dose | | | Confidential | | 2 | | Trastuzumab | | 4.02 | | 3 | | |
| PET normal | | | Confidential | | 2 | | Vinorelbin | | 2.45 | | 3 | | |
| PICC catheter | | | Confidential | | 2 | | X-ray | | Confidential | | 2 | | |
| Pleura drainage | | | 143.24 | | 1 | | Zoledronic acid | | 34.98 | | 3 | | |
| Policlinic visit | | | 134.24 | | 1 | |  | |  | |  | | |
| 1 | | Ref:  Institute for Medical Technology Assessment. BIJLAGE 1 Kostenhandleiding: Methodologie van kostenonderzoek en referentieprijzen voor economische evaluaties in de gezondheidszorg. Erasmus Universiteit Rotterdam. 2016. | | | | | | | | | | |  |
| 2 | | Maastricht University Medical Centre+. Internal cost prices. 2018. Confidential | | | | | | | | | | |  |
| 3 | | Zorginstituut Nederland. Medicijnkosten. 2018. Available at: https://www.medicijnkosten.nl/ | | | | | | | | | | |  |
| 4 | | Franken MF, Kanters T, Uyl-de Groot C. Insights into healthcare and societal costs of subcutaneous injection and intravenous infusion of trastuzumab for HER2 positive breast cancer and rituximab for non-Hodgkin’s lymphoma in The Netherlands. 2017. Available at: https://www.imta.nl/assets/uploads/2017/12/Abstract-IMTA_2017_1205.pdf | | | | | | | | | | |  |
| 5 | | Essers BA, Seferina SC, Tjan‐Heijnen VC, Severens JL, Novák A, Pompen M, Oron UH, Joore MA. Transferability of model‐based economic evaluations: the case of trastuzumab for the adjuvant treatment of HER2‐positive early breast cancer in the Netherlands. Value in health. 2010 Jun 1;13(4):375-80. doi: 10.1111/j.1524-4733.2009.00683.x | | | | | | | | | | |  |
| 6 | | Timmer-Bonte, JN, Adang, EM, Smit, HJ et al. Cost-effectiveness of adding granulocyte colony-stimulating factor to primary prophylaxis with antibiotics in small-cell lung cancer. J Clin Oncol. 2006; 24: 2991–2997 | | | | | | | | | | |  |
