# Supplementary material for: Variability of cost trajectories over the last year of life in patients with advanced breast cancer in the Netherlands
Source: PLoS One. 2020 Apr 9;15(4):e0230909. doi: 10.1371/journal.pone.0230909 (PMC7145011; doi:10.1371/journal.pone.0230909)
Supplement: S2 Table — (DOCX) [file pone.0230909.s002.docx]

| **S2 Table: Final zero-inflated Poisson model – Beta coefficient point estimates** | | | | |
| --- | --- | --- | --- | --- |
| **Count model coefficients (Poisson with log link)** | | | | |
|  | Intercept | Poly 1 | Poly 2 | Poly 3 |
| MCI | 7.872* | -0.368* | 0.096 | -0.005 |
| HSD | 8.239* | 0.365 | -0.067 | 0.003 |
| MFEM | 7.426* | 0.584 | -0.130 | 0.007 |
| MFLM | 8.459* | -0.833 | 0.180 | -0.010 |
| LSPE | 6.101* | 0.580* | -0.122* | 0.008 |
| LSL | 6.408* | -0.189* | 0.031 | -0.001 |
|  |  |  |  |  |
| **Zero-inflation model coefficients (binomial with logit link)** | | | | |
|  | Intercept | Poly 1 | Poly 2 | Poly 3 |
| MCI | 0.432 | -0.478 | 0.087 | -0.006 |
| HSD | 0.019 | -0.256 | 0.036 | -0.002 |
| MFEM | -0.343 | -0.172 | 0.011 | -0.001 |
| MFLM | -0.120 | -0.161 | 0.029 | -0.003 |
| LSPE | 0.561 | -0.561* | 0.116* | -0.008* |
| LSL | -0.551 | -0.070 | -0.010 | 0.000 |
