# Supplementary material for: Variability of cost trajectories over the last year of life in patients with advanced breast cancer in the Netherlands
Source: PLoS One. 2020 Apr 9;15(4):e0230909. doi: 10.1371/journal.pone.0230909 (PMC7145011; doi:10.1371/journal.pone.0230909)
Supplement: S3 Table — (DOCX) [file pone.0230909.s003.docx]

| **S3 Table: Zero-inflated Poisson model – Beta coefficient point estimates for the subgroup of patients with at least 12 months survival time (n=324)** | | | | |
| --- | --- | --- | --- | --- |
| **Count model coefficients (Poisson with log link)** | | | | |
|  | Intercept | Poly 1 | Poly 2 | Poly 3 |
| G1 | 8.393* | -0.787* | 0.169 | -0.008 |
| G2 | 8.495* | 0.158 | -0.033 | 0.002 |
| G3 | 7.109* | -0.435 | 0.122 | -0.008 |
| G4 | 6.317* | 0.329* | -0.074 | 0.005 |
| G5 | 7.698* | 0.422 | -0.098 | 0.005 |
| G6 | 6.512* | -0.366* | 0.035 | -0.001 |
|  |  |  |  |  |
| **Zero-inflation model coefficients (binomial with logit link)** | | | | |
|  | Intercept | Poly 1 | Poly 2 | Poly 3 |
| G1 | -28.223* | 8.665* | -1.011* | 0.039 |
| G2 | -4.112* | 0.182 | -0.020 | 0.001 |
| G3 | -11.460* | 1.358 | 0.069 | -0.013 |
| G4 | -3.772* | -0.311 | 0.096 | -0.006 |
| G5 | -4.460* | 0.069 | 0.033 | -0.002 |
| G6 | -1.983* | 0.168 | -0.039 | 0.002 |
