# Supplementary material for: Variability of cost trajectories over the last year of life in patients with advanced breast cancer in the Netherlands
Source: PLoS One. 2020 Apr 9;15(4):e0230909. doi: 10.1371/journal.pone.0230909 (PMC7145011; doi:10.1371/journal.pone.0230909)
Supplement: S4 Table — (DOCX) [file pone.0230909.s004.docx]

| **S4 Table: Latent group allocation agreement matrix.** Full cohort versus the subgroup analysis in patients with at least 12 months of survival time. | | | | | | | |
| --- | --- | --- | --- | --- | --- | --- | --- |
| **Full cohort / subgroup** | **G1** | **G2** | **G3** | **G4** | **G5** | **G6** | **n (+ patients with <**  **12months survival)** |
| **MCI** | 20 | 0 | 0 | 0 | 0 | 0 | 20 (+21) |
| **HSD** | 0 | 35 | 1 | 0 | 0 | 3 | 39 (+28) |
| **MFEM** | 0 | 0 | 48 | 11 | 0 | 0 | 59 (+38) |
| **MFLM** | 18 | 4 | 15 | 17 | 2 | 0 | 56 (+45) |
| **LSPE** | 0 | 0 | 1 | 0 | 50 | 0 | 51 (+56) |
| **LSL** | 0 | 0 | 0 | 27 | 3 | 69 | 99 (+46) |
| **n** | 38 | 39 | 65 | 55 | 55 | 72 |  |
| **Weighted Cohen’s kappa (95%CI)** | 0.79 (0.78 – 0.80) | | | | | | |
