# Supplementary material for: Variability of cost trajectories over the last year of life in patients with advanced breast cancer in the Netherlands
Source: PLoS One. 2020 Apr 9;15(4):e0230909. doi: 10.1371/journal.pone.0230909 (PMC7145011; doi:10.1371/journal.pone.0230909)
Supplement: S1 File — (DOCX) [file pone.0230909.s005.docx]

**S1 File: Handling of missing data and vague time information**

As described in the main article, as a consequence of retrieving routine data from real world patients, we faced different missing data problems.

First, the SONABRE registry contains information on how many units of certain hospital resources were consumed by the patients during consecutive treatment sequences. In some instances (0.9% of data fields and 51 (9.1%) patients), the quantity of resource consumption was unknown. This was mostly a problem in medicines, for which missingness was limited to the dosage that was given, while the type of drug and the number of administrations were reported. In order to avoid the creation of implausible observations, we used hot deck imputation to replace missing values with observed values from another patient. In the absence of other relevant predictors, patients were matched only on the particular resource for which consumption had to be imputed.

Second, for some hospital resources, the exact dates of resource consumption were not registered in the database. The available data included information on the first and last day of systemic treatments, the dates of surgical and radio-therapeutic procedures, as well as hospital admissions. Other resources, especially diagnostic procedures, lacked granular date information, so that costs could not be assigned to specific dates, but had to be attributed to consecutive 'treatment sequences', which were defined as the period between the start of a systemic treatment and a change in the combination of agents, or the start of a new next systemic treatment, or death. Sequences can include treatment-free intervals. If patients did not receive any systemic therapy, their first treatment sequence continues until death.

Third, in some cases, the consumption of resource units was known, but the dates of consumption (either exact dates, or start/stop dates, or treatment sequence start/stop dates) were missing. Here, we used another approach to replace the missing values. In order to avoid creating artificial patterns, the following rules were applied: 1. If the start date of resource use was missing, the date was set to the date of diagnosis (of advanced disease); 2. if the end date was missing, it was set to the day of death or censoring; 3. If both start and end dates were missing, the costs of resources were distributed over the entire patient follow-up time; 4. If only the day of the month was missing, it was set to the 15th of the month. In cases in which this resulted in implausible time information (i.e. treatment started before diagnosis or stopped after death), the missing day was set as specified above.

Fourth, for endocrine therapies and bisphosphonates, the registry did not contain information on the number of administrations and dosages, but on treatment duration. Consumption was then computed by multiplying the time on treatment with a standard dose regime. When treatment durations of bisphosphonate or endocrine treatments were missing (start and/or stop dates were not reported) treatment durations were imputed using the average proportion of survival time on treatment (and not the imputed start/stop dates, which would have resulted in an overestimation).

Finally, for 40 patients, the HER2 and/or HR receptor status was unknown, either because it was not tested, or results were not reported in the electronic health records. These patients were classified as ‘negative’, which also matched the treatment regimens they received.
