# Supplementary material for: Variability of cost trajectories over the last year of life in patients with advanced breast cancer in the Netherlands
Source: PLoS One. 2020 Apr 9;15(4):e0230909. doi: 10.1371/journal.pone.0230909 (PMC7145011; doi:10.1371/journal.pone.0230909)
Supplement: S1 Fig — Lower values indicate better model fit. For nine groups, the model did not converge for polynomials greater 1. (DOCX) [file pone.0230909.s006.docx]

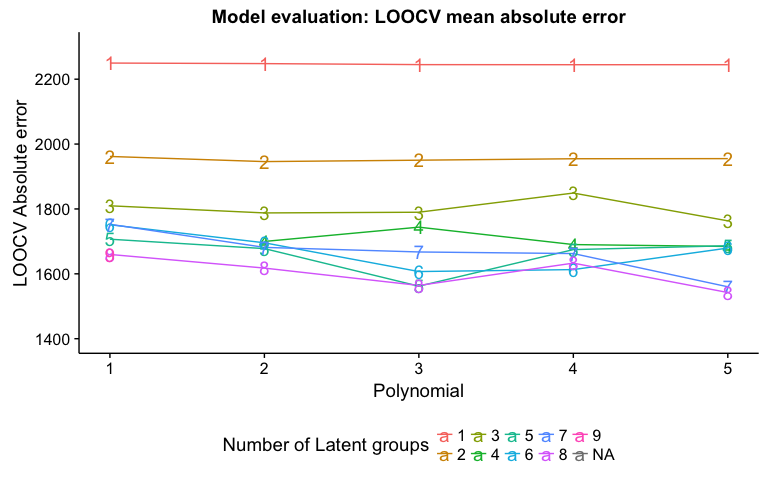


**S1 Fig**: The leave-one-out cross-validation (LOOCV) mean absolute error for fitted models with different numbers of latent groups (1-9) and different degrees of polynomials (1-5). Lower values indicate better model fit. For nine groups, the model did not converge for polynomials greater 1.
