# Supplementary material for: Variability of cost trajectories over the last year of life in patients with advanced breast cancer in the Netherlands
Source: PLoS One. 2020 Apr 9;15(4):e0230909. doi: 10.1371/journal.pone.0230909 (PMC7145011; doi:10.1371/journal.pone.0230909)
Supplement: S4 Fig — For convenience, each subgroup was colour-matched to the subgroup from the full cohort analysis, to which it was most similar. The top plot shows the results of the fitted GBTM model with six latent cost trajectory groups and cubic polynomials. For comparative purposes, the overall average trend is also shown. Below, the mean trajectories for each latent group (observed = dotted, estimated = solid line), in combination with the observed trajectories of the individual patients are presented. (DOCX) [file pone.0230909.s009.docx]

**
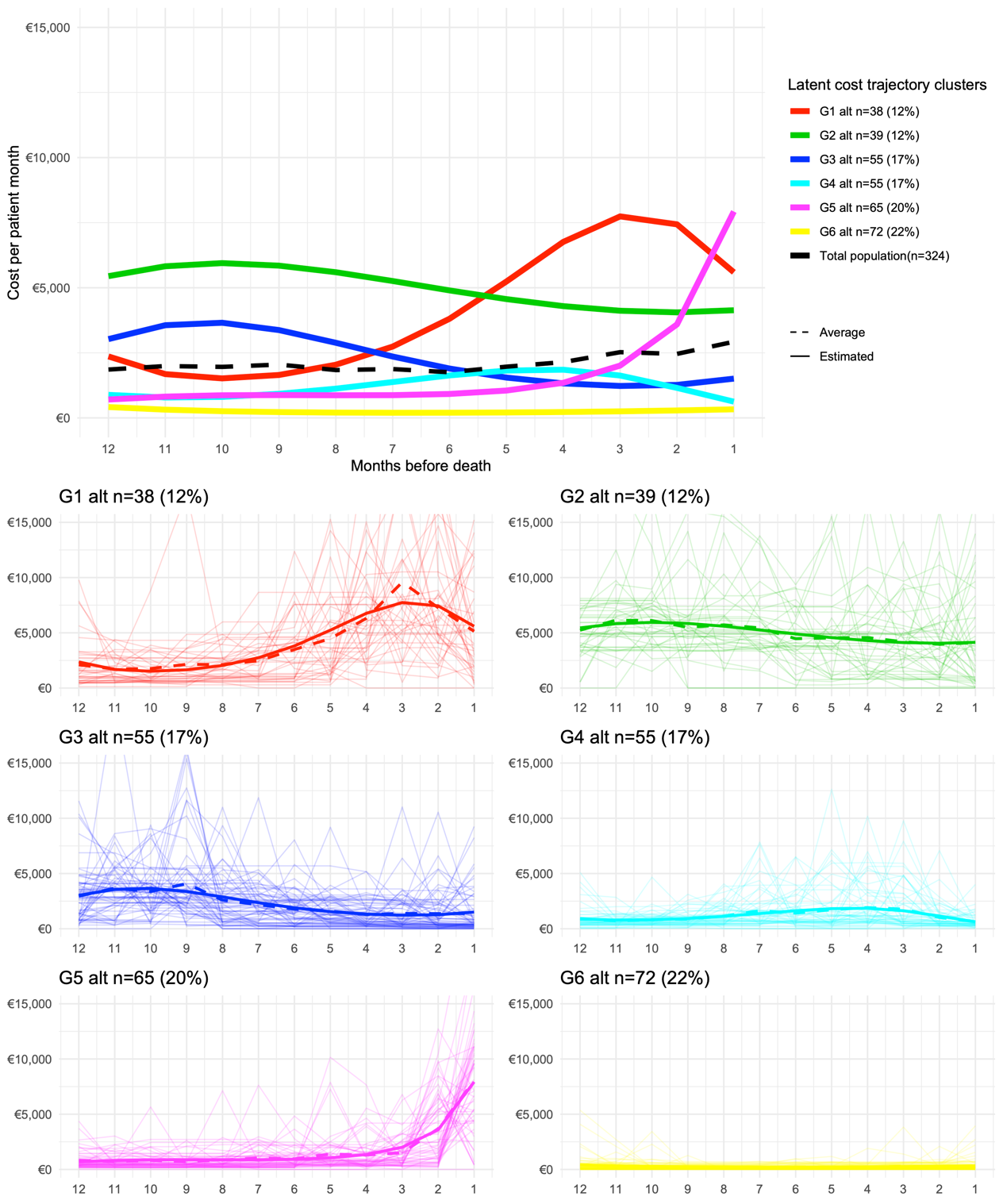
**

**S4 Fig**: Group-based trajectory modeling – Latent cost trajectory groups in the subgroup of patients with at least twelve months survival time (n=324). For convenience, each subgroup was colour-matched to the subgroup from the full cohort analysis, to which it was most similar. The top plot shows the results of the fitted GBTM model with six latent cost trajectory groups and cubic polynomials. For comparative purposes, the overall average trend is also shown. Below, the mean trajectories for each latent group (observed = dotted, estimated = solid line), in combination with the observed trajectories of the individual patients are presented.
