# Supplementary material for: Variability of cost trajectories over the last year of life in patients with advanced breast cancer in the Netherlands
Source: PLoS One. 2020 Apr 9;15(4):e0230909. doi: 10.1371/journal.pone.0230909 (PMC7145011; doi:10.1371/journal.pone.0230909)
Supplement: S6 Fig — Results for the subgroup of patients with at least twelve months survival time. Lower values indicate better model fit. For six groups, the model did not converge for polynomials greater 3. (DOCX) [file pone.0230909.s011.docx]

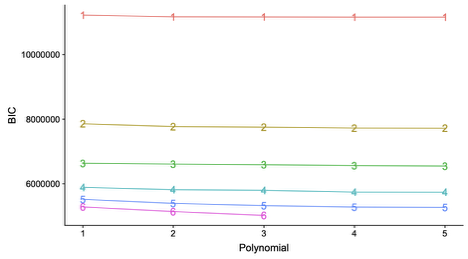


**S6 Fig**: The Bayesian information criterion (BIC) values for fitted models with different numbers of latent groups (1-9) and different degrees of polynomials (1-5). Results for the subgroup of patients with at least twelve months survival time. Lower values indicate better model fit. For six groups, the model did not converge for polynomials greater 3.
