# Supplementary material for: Variability of cost trajectories over the last year of life in patients with advanced breast cancer in the Netherlands
Source: PLoS One. 2020 Apr 9;15(4):e0230909. doi: 10.1371/journal.pone.0230909 (PMC7145011; doi:10.1371/journal.pone.0230909)
Supplement: Attachment — Submitted filename: Response to reviewers.docx [file pone.0230909.s012.docx]

**Response to Reviewers' comments** (for publication)

| **Editor** | | |
| --- | --- | --- |
| 1 | 1. Please ensure that your manuscript meets PLOS ONE's style requirements, including those for file naming. […] | Manuscript has been reformatted accordingly |
| 2 | 2. We note that you have indicated that data from this study are available upon request. […]  In your revised cover letter, please address the following prompts:  a) If there are ethical or legal restrictions on sharing a de-identified data set, please explain them in detail (e.g., data contain potentially identifying or sensitive patient information) and who has imposed them (e.g., an ethics committee). Please also provide contact information for a data access committee, ethics committee, or other institutional body to which data requests may be sent.  […] | *Please update our data availability statement as follows:*  *The data contains potentially identifiable patient information. It cannot be shared publicly due to ethical and legal restrictions. However, the data of the SONABRE registry is organized according to the FAIR principles and researchers who meet the criteria for access to confidential data can submit their requests to the registry’s PI prof. V.C.G. Tjan-Heijnen (*[*vcg.tjan.heijnen@mumc.nl*](mailto:vcg.tjan.heijnen@mumc.nl)*).* |
|  |  |  |
| 3 | 3. [..]  We note that you received funding from a commercial source: Novartis, Pfizer, Roche Nederland B.V., Eisai B.V., and Eli Lilly N.  a. Please provide an amended Competing Interests Statement that explicitly states this commercial funder, along with any other [relevant declarations](http://www.plosone.org/static/competing.action) relating to employment, consultancy, patents, products in development, marketed products, etc.  […]  b. Please include your amended Competing Interests Statement within your cover letter. We will change the online submission form on your behalf. | We provide an amendment for the Competing Interests Statement below. We would be grateful if you could change the online submission form on our behalf.  *The data collection of the Southeast Netherlands Advanced Breast Cancer Registry was funded by the Netherlands Organization for Health Research and Development (ZonMw), Novartis, Pfizer, Roche Nederland B.V., Eisai B.V., and Eli Lilly NL. The funders had no role in study design, data collection and analysis, decision to publish, or preparation of the manuscript, and this does also not alter our adherence to PLOS ONE policies on sharing data and materials.* |
| 4 | 4. Please include captions for your Supporting Information files at the end of your manuscript, and update any in-text citations to match accordingly. […] | We added a supplementary information section at the end of the manuscript and split the content of the appendix into separate files. |
| **Reviewer #1** | | |
| 5 | This research is a novel approach to the costing of end of life costs. This is an important research reminding that there are many different and so far unexplained cost-trajectories which are not considered by conventional health state-based cost-effectiveness analyses. The paper was written with scientific rigor as well as with an enjoyable flow. |  |
| 6 | There is a growing body of literature regarding the determinants of end-of-life costs and the readers could potentially benefit from some additional discussion of the literature although I do not deem this as a necessity. This research is an important contribution to the analysis of healthcare costs. | The literature on the wider determinants of (high) end-of-life costs is indeed interesting. However, a general discussion of the research on this topic is outside the scope of this paper. We thus limit ourselves here to adding a short note the introduction section:  *The general trend of an increase in health care costs towards the end of life is not limited to ABC patients, but also widely observed in other patient populations [5, 7]* |
| **Reviewer #2** | | |
| 7 | Strength of this article  The significance of this article is that it goes beyond the average trajectory and explores subgroups with distinct trajectory patterns. Such patterns are often lost in an analysis that focuses on average trajectory.  The group-based trajectory modeling has been used in the literature and it is a useful method to explore the different trajectory patterns. |  |
| 8 | Weakness of this article  The sample size (n = 558) is a little bit small for this type of studies. Large datasets can often be obtained from, for example, insurance claims database, and breast cancer is not a rare disease among cancer patient population. | As a rule of thumb, researchers may regard a sample size of N ~ 500 as small to moderate when handling mixture models. The caution is required because mixture models have many parameters to be estimated, even more so when several classes are extracted. Sample size, however, is not the most prominent data feature that affects model performance. Lager N’s are desirable, but can just as well be useless if the degree of class-separation in the data is low (no marking heterogeneity in longitudinal paths). By contrast, if the latter is present, smaller Ns~ 100/200 have already been shown to yield solid latent trajectories. On the grounds of the unveiled heterogeneous picture of cost trajectories in our study, we are confident that the sample size sufficed for class-extraction. While we would agree that, all else being equal, using larger data sets would be beneficial to our analysis, we would argue that even though administrative data (e.g. insurance claims) could provide more data points, the quality of the data is likely to be much poorer. We consider it a particular strength of our study that clinical characteristics and cost estimates are based on information retrieved from hospital records. |
| 9 | There is a little bit lack of depth in this article, because the possible reasons for the different trajectory shapes are not studied (I recognize that this is left to future work, but it does diminish the depth of this paper to some degree). | GBTM analyses are generally conducted in two steps: First, the model is selected (i.e. the number of trajectories and their individual shapes determined); second, the extracted trajectories are ‘validated’ (similar to the concept of construct validation is questionnaire analysis). The process is a regressive form of inference: longitudinal paths are first extracted, and how they came about is subsequently studied by exploring their potential shapers and triggers.  In our paper, we started with the identification of cost trajectories, and, as a second step, sought to understand patients’ individual features that could explain their distinct longitudinal paths. See tables 2 and 3 of trajectories profiling and the multinomial logistic model, where we explored the connections between clinical and treatment characteristics to trajectory membership. We would thus argue that we did, in fact, made a first step and studied the reasons for the different trajectory shapes. Nevertheless, we agree with the reviewer that it would indeed be interesting for future studies to expand the analysis of the potential and investigate those in more detail.  We revised the final paragraph of the discussion section (also see response to comment #12):  *GBTM proved to be a promising and useful tool for exploring latent patterns in the cost data of ABC patients. To what degree the approach can lead to actionable insight will be for future studies to determine. Those could build on our results and should aim to explain what causes the longitudinal patterns of costs. Even if differences in cost trajectories cannot (yet) be easily deduced from clinical characteristics, economic evaluations of treatments in ABC patients should aim to account for the heterogeneity of costs between and within patients over time.* |
| 10 | Even without looking at the conclusion of this article, one can speculate that each patient's cost trajectory is more or less different because of various patient-level, physician-level and hospital-level factors. Therefore, heterogeneity in cost trajectory exists. | Our point exactly! This multitude of (known and unknown) factors independently or jointly affecting the outcome is one of the main ideas driving the applications of GBTM or mixture models. These models are referred to as persons-centered approaches (as opposed to variables-centered), because they take a more holistic point of view. For clarity: in traditional longitudinal models, like mixed model, longitudinal variability of an outcome is decomposed and attributed to different sources of variation, the explanatory variables (i.e. variables-centered). Persons-centered approaches, by contrast, conceptualize individuals as an integrated whole, instead of the sum of its parts. For this reason, GBTM is particularly useful when emergent patterns (e.g. latent trajectories) are considered indivisible, because the difficulty of decomposing the total into the sum of individual components (explanatory variables) and their interactions. |
| 11 | The number six, i.e., six pattern groups, is perhaps more or less subjective. If one has a much larger dataset, perhaps more patterns can be identified by the statistical criteria used in this paper. | We would maintain that GBTM model selection is by no means arbitrary – There are formal statistical criteria to guide the process [see the section “Identification of latent cost trajectories” where we laid out the analysis plan and which model fit criteria were employed]. However, because model fit indices are not always watertight, and are often inconclusive, researchers often have to resort to heuristic strategies and substantive interpretation to come to a final model. We are transparent about this point and report model fit statistics in the appendix. With regard to the point that other, larger datasets might yield additional latent trajectories, we have added an additional clarification in the discussion – also in response to comment #23:  *[…] This means that even though we think that the application of GBTM in similar settings (e.g. health insurance claims data, or ABC patients from other regions) will lead to similar conclusions, it is unlikely that the exact same latent trajectory groups will be uncovered.* |
| 12 | Without looking into the inside mechanism that leads to the different patterns, the conclusion and results of this article is superficial. | If we may, we respectfully disagree with the reviewer. As explained in response to comment #9, class-profiling (table 2) and multinomial logistic models results (table 3) served exactly the purpose to explore the potential inside mechanisms generating the trajectories’ variability. That the available variables were only weakly predictive of trajectory membership was unfortunate, indeed, but nonetheless an important finding! The sizes of the respective latent groups might also be informative.  That being said, we share the reviewers’ sentiment to demand further research. We thus revised the final paragraph of the discussion section:  *GBTM proved to be a promising and useful tool for exploring latent patterns in the cost data of ABC patients. To what degree the approach can lead to actionable insight will be for future studies to determine.Those could build on our results and should aim to explain what causes the longitudinal patterns of costs. Even if differences in cost trajectories cannot (yet) be easily deduced from clinical characteristics, economic evaluations of treatments in ABC patients should aim to account for the heterogeneity of costs between and within patients over time.* |
| 13 | The conclusion says: "From this average trend, however, no inferences can be made with respect to individual patients or subgroups of patients." I am skeptical about this statement. This paper identified six trajectory shapes. For an individual, certainly we can plot the cost trajectory of that person and determine which shape group this person's trajectory most likely belongs to. | This comment raises two distinct issues:  1. There might be a minor misunderstanding. Our paper makes the point, hopefully cogently, that a researcher cannot gauge cost trajectories of individual patients based on this overall (one-model fits all) average population trend. We were able to show that individual departures from the total average were considerable in degree and kind. The sentence has thus to be interpreted the other way around: the overall average cost trajectory does not tell much about the cost trajectory of any particular patient. One may simply take this as a reminder of the ‘ecological inference fallacy’. To avoid any confusion, we have revised the respective sentence:  *This demonstrates that average population trend should not be mistaken as the cost trajectory of ‘an average patient’ – it cannot be used to make inferences about individual ABC patients, or even subgroups of patients [21].*  2. It is true and central to the objective of the paper that we ”can plot the cost trajectory of [an individual] and determine which shape group this person's trajectory most likely belongs to”. Once a final model is selected, GBTM computes posterior probabilities of group membership for each individual. (S)he is then assigned to the group based on his/her highest posterior group probability. We have added the following sentence to the methods section to clarify this point:  *The basic rationale is to cluster subjects in groups that maximizes both within-groups commonalities and between-groups differences.* |
| 14 | The subsection "Longitudinal Patterns in Costs During the Last Twelve Months Before Death" says: "If the time between ABC diagnosis and death was shorter, patients only contributed costs to the months in which they were diagnosed and alive (i.e. only to the last x months)." I disagree with this way of handling patients with less than 12 month of follow-up. It is better to focus only on patients with at least 12 months of follow-up. It is difficult to justify averaging over patients with different lengths of follow-up. | While we acknowledge that averaging over patients with different survival times is not ideal, we think that there is no better alternative. In our analysis, a total of 234 (42%) patients had a survival time of less than 12 months. Excluding those patients would not only significantly reduce the sample size, but it might also bias the results: advanced breast cancer patients with short survival times might have more aggressive forms of disease than patients who lived longer and/or they might be non-responders to systemic treatments. These differences might cause specific temporal patterns of health care usage and spending, which we would like to capture in our analysis.  The reviewer’s comment, in combination with comment #24, nevertheless, led us to investigate whether the extracted latent cost trajectory patterns could actually be in part an artefact, caused by aggregating costs of patients with different survival times. We re-ran the GBTM analysis, only including patients with at least 12 months of survival time. We found that the latent trajectory groups extracted from this selected patient group were very similar to the ones extracted from the full cohort. Please see Appendix S7-10 for further details. We take this as evidence for the robustness of our findings.  The results of our subgroup analysis are provided in the appendix and the following sentences were added to the manuscript:  Methods/Identification of latent cost trajectories:  *Since the aggregation of costs across patients with different survival times could, potentially, create artificial patterns, we conducted a sensitivity analysis in the subgroup of patients with at least 12 months survival time. Latent cost trajectory groups were extracted analogous to the methodology used in the full cohort model (for efficiency reasons, LOOCV was not conducted and model selection was based on AIC and BIC alone). Results are provided in Appendix S7-10.*  Results/Identification of latent cost trajectories:  *A sensitivity analysis in the subgroup of patients with at least twelve months survival time (n=324) was conducted. Six latent cost trajectory groups were extracted, which were very similar to the groups found in the full cohort model. With a weighted Cohen’s kappa of 0.79, the class allocation agreement was judged to be substantial. However, the peak in costs in the last month before death was less pronounced, and minor differences in latent trajectory patterns were observed. The detailed results are in Appendix S7-10. Overall, the results of the sensitivity analysis indicate that trajectory extraction was not materially affected by missingness.*  Discussion:  *[…] In a subgroup analysis, we found these results to be robust: similar latent cost trajectory patterns were uncovered in the subgroup of patients with at least twelve months of survival time.* |
| 15 | Clarification needed: The costs are aggregated by month. If a patient dies in the middle of the month, then the last month is not a full month, which drives down the cost. Please explain how this issue was handled. | We are grateful for this thoughtful question – First of all, we would like to note that every patient, by definition, died after the last day of the last month before death. The only month for which not all days can be observed is the one in which the patient enters the study (e.g. 10 months before death). We state in the paper:  *If the time between ABC diagnosis and death was shorter, patients only contributed costs to the months in which they were diagnosed and alive (i.e. only to the last x-months).*  To the second part of the question, how we handled costs of an incomplete month: We computed monthly costs as the sum of the costs incurred on each day. This was the only plausible approach, because we also estimated and reported absolute end-of-life costs and average monthly costs for different types of resources. It is true that, as a result of our aggregation method, the costs of the first month of any patient might be lower, but the method was consistently applied to all patients. We have revised the respective section in the method section to be more transparent about this point:  *We assessed the average trends in monthly hospital costs of ABC patients over the last twelve months before death. If the time between ABC diagnosis and death was shorter, patients only contributed costs to the days and months in which they were diagnosed and alive (e.g. only to the last 15 days and 9-months). The costs of partially observed months (e.g. 15 days) following the diagnosis of ABC reflect the unadjusted sum of the daily costs.* |
| 16 | Please explain how the time-stamp of the costs is determined. If a procedure is performed on a patient, did you attribute the cost to the date on which the procedure is performed, or to the date when the billing for that procedure is processed? If medication is prescribed, did you attribute the medication cost to the date of prescription or distribute the medication cost across all the days in the prescription period? | Our study is based on data extracted from hospital patient records, so that we were in the fortunate position to be able to attribute the costs of most resources to the days on which they were consumed. For most medications, data was available on the quantity that was prescribed, the first and the last day of prescription, and the number of administrations. For simplicity, costs were distributed equally between the first and the last day. We revised and expanded the beginning of the respective section (see below) and restructured the following paragraph to better explain how the data were handled.  *Whenever possible, we aimed to allocate costs to the days on which resources were consumed. Costs of systemic treatments were equally distributed between the first and the last day of administration. Because our study was based on routinely collected data, missingness was unavoidable. In some cases, the times of consumption were (partially) missing, and for certain resources, the exact dates of consumption were not registered and had to be inferred. This was mainly the case for diagnostic procedures, for which granularity of time information was limited. Respective costs could not be assigned to specific dates, but had to be attributed to consecutive treatment sequences. Moreover, in some instances, the quantities of resource consumption were unknown and had to be imputed [11]. A detailed description of the employed methods is provided in Appendix S2 in the Electronic Supplementary Material.* |
| 17 | Page 14 of the paper says "We fitted the cost data of ABC patients using zero-inflated Poisson models, to account for excess zeros". Further details need to be provided about this model (maybe in appendix). First, how do you justify this is zero-inflated Poisson instead of the usual Poisson model? | The choice to use a zero-inflated poisson (ZIP) model was partly predefined by the decision to conduct the GBTM using R – the crimcv package implements a ZIP and does not allow for other link functions. It might have been feasible to adjust the code of the package, or to use a different statistical program, however, a ZIP model also seemed conceptually well suited, and hospital costs are commonly modeled using a ZIP. The theory is that there are two separate statistical processes: one determines whether a patient develops a health care need (progression of disease, complications etc), or not, which would result in, so called structural zeros. The other process determines the costs of hospital care, conditional on that the patient has developed a health problem. |
| 18 | Second, how did you deal with overdispersion, a common difficulty with Poisson model? | In mixture models, overdispersion is, in general, not a problem. In fact, because mixture models allow for greater variability than one-parameter distributions – they are a mix of several distributions - they are particularly suited for analysis of taxing types of data, characterized by e.g. violations of normality and/or overdispersion [see e.g. Choongrak Kim (1994) Dispersion statistics in overdispersed mixture models, Communications in Statistics - Theory and Methods, 23:1, 27-45] |
| 19 | Third, this could be longitudinal zero-inflated Poisson, and accommodating the longitudinal nature of data (i.e., intra-subject correlation among the 12 monthly costs per patient) is not a computationally trivial task. How did you handle this situation in the software that you use? It would be better to present the most important model information without asking the reader to read the documentation of crimCV package. | The reviewer pertinently raises the issue of the correlations among repeated measures. How this is dealt with in mixture models, however, is not a straightforward matter, and may require some a balancing act. The predicament is as follow: Given the abundance of parameters, one important assumption of mixture modeling techniques, like the GBTM, is that correlations among (repeated) measures are fully captured by the latent classes. In our cost trajectories model, this means that *conditional* on the final class-assignment, subjects’ deviations from their trajectory-specific average are solely (random noise) error variability. On top of that, error variance is often set to be equal over all classes (class invariant). This is a set of rigid assumptions, for sure. Some mixture models can relax this (class-invariant) conditional independence by considering random effects and/or auto-correlated errors, and/or even allow error variance to change over classes. But here, the researcher needs to negotiate a trade-off between model’s increased complexity and convergence problems. The crimCV package also does not allow for testing different covariance structures, the default one being conditional independence. Even for this simple approach, the computational challenge became apparent when the model failed to converge with 9+ classes and degree 5 polynomials. To estimate the models (for 1-5 degree polynomials and 1-9 latent groups) the analysis ran for several hours on a conventional high-spec computer. In spite of this long explanation, we would like to wrap up this reaction by quoting one of the developers of GBTM (Daniel Nagin) in his defense of the simpler models: “The trajectory groups are constructs for approximating a more complex underlying structure… Despite being an exercise of approximation (point supports in an unknown population distribution), it can still be used for valid statistical inferences”. |
| 20 | Page 14. Please clarify the time of measurement for some covariates: age, co-morbidity, and type of ABS treatment (what if the treatment changes over time?). | We have revised the respective part of the method sections:  *The following characteristics were available and considered relevant: age, survival time (time between the date of ABC diagnosis and death), metastatic sites (any time), initial HR and HER2 receptor status, and chronic comorbidities (other malignancy, metabolic, cardio-vascular, or pulmonary disease, diagnosed at any time), as well as the last type of ABC treatment that was administered (chemo-, hormonal-, and/or targeted therapy; locoregional radical).* |
| 21 | Please also clarify how the survival time is calculated if every patient has 12 month follow-up. | See the response to comment (#20) above:  “[..] survival time (time between the date of ABC diagnosis and death) [...].” |
|  |  |  |
| **Reviewer #3** | | |
| 22 | Schneider et al. analyzed health care utilization data of 558 advanced breast cancer cases in the Netherlands using group-based trajectory modelling. The authors noted interesting patterns. While it is generally assumed that end-of-life health care expenditures follow a hockeystick-like trajectory, their analysis revealed six distinct patterns with very differing shapes. Of note, only two out of the six patterns showed an increase towards the final life months. The patterns were largely driven by medication costs (until around month 6) and later by hospitalization (possibly indicating a switch of care focus to palliative care).  The analysis is generally sound and the paper is well written. I also like the approach the authors have undertaken: away from mean-based comparisons toward more comprehensive assessments of longitudinal costs. That said, I have three major comments for the author's consideration. |  |
| 23 | 1) The cost data are not actual costs from healthinsurance claims but derived from health care use (specific medications, procedures), with acutal costs derived and aggregated from official sources. This has the advantage that one does not need to worry about inflation when analyzing multiple years. But this has also two downsides: costs accrued outside the hospital setting (e.g. palliative care) are not included (which is acknowledged in the discussion), but also a lot of the variability observed in claims data is removed. This latter point is particularly important here. It leads me to believe that the findings will likely be not reproducible when applying the same methodology to health care expenditure data. In fact, their finding of six distinct and quite robust groups may even be overly optimistic. Real-world (claims) data is messy and noisy. Therefore I would be surprised if the method performed equally well in other databases of similar size. | It is quite likely that we may find different patterns in different data and contexts. The number of extracted trajectories, their sizes, levels and shapes, even if the same outcome is considered, can be expected to vary across studies. This may have several reasons, but an important point to consider is that latent trajectories are neither immutable nor true entities. Interpreting them as such would amount to reifying them (the fallacy of reification). Instead, as a general rule, they should be regarded and distinctive longitudinal features of the data. The reviewer’s point is nonetheless valid and we have amended the respective part in the discussion section:  *Parsimony and the interpretability of the extracted trajectories also played a role in model building. This means that even though we think that the application of GBTM in similar settings (e.g. health insurance claims data, or ABC patients from other regions) will lead to similar conclusions, it is unlikely that the exact same latent trajectory groups will be uncovered.* |
| 24 | 2) I am a little concerned about censoring due to death in the analysis. More than 40% of patients died in less than 12 months after diagnosis, which could have influenced trajectory shapes (e.g. the HSD and MFEM groups, which have high costs in the first three months after diagnosis). In other words, the observed trajectory patterns could somehow also originate from data availability. Does the statistical method offer possibilities to include censoring? If not, are there other means to verify that the observed groups are not artifacts of follow-up duration or missing data? | We think there are several important issues that the reviewer’s comment touches on.  1. A minor clarification: HSD and MFEM have high costs in months 12-9 before death, not “in the first three month after diagnosis” – although, for a few patients, these periods might have coincided, of course.  2. As discussed above (see response to comment #14), for patients with survival times of less than 12 months, the costs of the first month in which they enter the study could be lower. The method we used to aggregate costs to the monthly level is not expected to result in higher costs though. If costs were high in patients who are newly diagnosed, we would by not consider this an artefact but a ‘true’ pattern in the data.  3. The notion of missing data might be also somewhat misleading in this context. If a patient had a survival time of, say, 10 months between the date of diagnosis and the date of death, costs for months 11 and 12 cannot be considered ‘missing’. Medical spending for advanced breast cancer can of course not occur before the diagnosis. Since we are not omitting some, potentially observable data points, results should not be considered *biased* by censoring or missing data.  4. It is true though that patients may be in different phases of their disease and the reviewer’s comment (in combination with comment # 14) led us to consider the possibility that the extracted latent cost trajectory patterns might indeed be some form of artifact. We re-ran the GBTM analysis, only including patients with at least 12 months of survival time. We found that the latent trajectory groups extracted from this selected patient group were very similar to the ones extracted from the full cohort. Please see Appendix S7-10 for further details. We take this as evidence for the robustness of our findings.  The results of our subgroup analysis are provided in the appendix and the following sentences were added to the manuscript:  Methods/Identification of latent cost trajectories:  *Since the aggregation of costs across patients with different survival times could, potentially, create artificial patterns, we conducted a sensitivity analysis in the subgroup of patients with at least 12 months survival time. Latent cost trajectory groups were extracted analogous to the methodology used in the full cohort model (for efficiency reasons, LOOCV was not conducted and model selection was based on AIC and BIC alone). Results are provided in Appendix S7-10.*  Results/Identification of latent cost trajectories:  *A sensitivity analysis in the subgroup of patients with at least twelve months survival time (n=324) was conducted. Six latent cost trajectory groups were extracted, which were very similar to the groups found in the full cohort model. With a weighted Cohen’s kappa of 0.79, the class allocation agreement was judged to be substantial. However, the peak in costs in the last month before death was less pronounced, and minor differences in latent trajectory patterns were observed. The detailed results are in Appendix S7-10. Overall, the results of the sensitivity analysis indicate that trajectory extraction was not materially affected by missingness.*  Discussion:  *[…] In a subgroup analysis, we found these results to be robust: similar latent cost trajectory patterns were uncovered in the subgroup of patients with at least twelve months of survival time.* |
| 25 | 3) Advanced breast cancer is a condition where treatment strategies are dependent of genetic markers (HER2, Hormone receptors). It seems therefore a bit counterintuitive to group patients by cost trajectories rather than by genetic subgroups (which is what most physicians would do). Especially since health care utilization and expenditures differ by genetic marker / treatment group. Although this analysis is a nice illustration of cost trajectory analysis, it is probably not the typical use case for this type of method. I would probably resort to trajectory analysis if no other established subgroup definitions are available. On the other hand, the observation that certain genetic marker combinations are indeed predominant in some trajectories could be considered some kind of a validation. Can the authors elaborate for which situations /databases / settings they consider their trajectory analysis method particularly suitable? | First of all, we would like to emphasize that the distinction between the variable- and the person-centered approach indeed lies at the heart of our paper. It might seem counterintuitive at first to group patients solely by their cost trajectories, instead of defining groups ex-ante, but, we think, a close consideration of the extracted classes supports our approach: the HER2/HR receptor status was only a significant predictor of group membership in the bivariate, but not in the multivariable model!  To stress the conceptual difference between both and to explain why the latter is of interest, we added the following paragraph in the discussion section, (the entire section was slightly re-structured to improve the flow of the text):  *It seems important to stress the difference in the conceptual approach: we did not intend to investigate to what extent the cost trajectories of, say, patients with HER2+ ABC differ from those of patients with HER2- ABC. Rather, we only looked at the cost trajectory data, in order to identify clusters of trajectories that are similar to each other. This allowed us to uncover hitherto hidden patterns of longitudinal costs in ABC patients. We found a considerable overlap of the cost trajectories of different ABC receptor subgroups: Patients with HER2+ and HER2-, for example, may share a very similar cost trajectory, and, even though it was a significant predictor in the bi- and multivariable analysis, the receptor status only explained a small proportion of the variance in the data.*  We agree with the reviewer that further research should follow from our study. However, as the first application of GBTM in this field of research, we would argue that our findings do have merit and are of interest to the research community on its own. We revised the final part of the discussion section to discuss the implications of our findings more critically and inform future research projects:  *Although immediate implications for practice are difficult to draw, there are lessons to be learned from our findings. [...] A better understanding of the longitudinal dynamic structure of costs in ABC patients might help to improve the quality of economic evaluations and support better decision making in health policy. GBTM proved to be a promising and useful tool for exploring latent patterns in the cost data of ABC patients. To what degree the approach can lead to actionable insight will be for future studies to determine. Those could build on our results and should aim to explain what causes the longitudinal patterns of costs. Even if differences in cost trajectories cannot (yet) be easily deduced from clinical characteristics, economic evaluations of treatments in ABC patients should aim to account for the heterogeneity of costs between and within patients over time.* |
